# Supplementary material for: Subsequent primary cancer incidence among cancer survivors in the United States, 1975–2019: An age–period–cohort analysis
Source: PLoS Med. 2026 Apr 28;23(4):e1005034. doi: 10.1371/journal.pmed.1005034 (PMC13123941; doi:10.1371/journal.pmed.1005034)
Supplement: S1 Supplemental Figures — Fig A: Observed and fitted age-specific incidence among females. Fig B: Observed and fitted age-specific incidence among males. (DOCX) [file pmed.1005034.s002.docx]

Supplemental Figures

| Figure A. Observed and Fitted Age-Specific Incidence Among Females |
| --- |
|  |

| Figure B. Observed and Fitted Age-Specific Incidence Among Males |
| --- |
|  |
