# Supplementary material for: Subsequent primary cancer incidence among cancer survivors in the United States, 1975–2019: An age–period–cohort analysis
Source: PLoS Med. 2026 Apr 28;23(4):e1005034. doi: 10.1371/journal.pmed.1005034 (PMC13123941; doi:10.1371/journal.pmed.1005034)
Supplement: Attachment — Submitted filename: Response to Editorial Comments.docx [file pmed.1005034.s008.docx]

**Manuscript title:** *Trends in Subsequent Primary Cancer Incidence Among United States Cancer Survivors, 1975–2019: An Age‑Period‑Cohort Analysis* **Manuscript ID:** PMEDICINE‑D‑25‑03472R2

(New title: *Subsequent Primary Cancer Incidence Among United States Cancer Survivors, 1975–2019: An Age‑Period‑Cohort Analysis*)

Requests from Editors:

GENERAL
1. Please confirm that your title complies with to PLOS Medicine's style. Your title must be nondeclarative and not a question. It should begin with main concept if possible. "Effect of" (or “Impact of”) should be used only if causality can be inferred, i.e., for an RCT. Please place the study design ("A randomized controlled trial," "A retrospective study," "A modelling study," etc.) in the subtitle (ie, after a colon).

Response: We appreciate the guidance on title formatting. We confirm that our revised title complies with PLOS Medicine’s style. We removed the “Trends in” per editorial teams comment about “trend” (comment 6 below).

2. Statistical reporting: Please revise throughout the manuscript, including tables and figures.
- Please report statistical information as follows to improve clarity for the reader, ""XX% (95% CI [XX,YY]; p</=)"".
- Please separate upper and lower bounds with commas instead of hyphens as the latter can be confused with reporting of negative values.
- Please repeat statistical definitions (HR, CI etc.) for each set of parentheses.

Response: We revised all statistical reporting throughout the manuscript, tables, figures, and supplements to follow this format.

3. Please ensure that all abbreviations are defined at first use throughout the text (including statistical abbreviations).

Response: We reviewed the entire manuscript and ensured that all abbreviations are defined at first use.

4. Please ensure that tables and figures, including those in supplementary files, are appropriately referenced in the main text.

Response: We confirmed that all tables and figures, including those in the Supporting Information, are referenced in the main text.

5. Please review your text for claims of novelty or primacy (e.g. 'for the first time' or ‘novel’) and remove this language.

Response: We have removed all novelty‑related language (e.g., in the first sentence of the Discussion and in the second limitation).

6. Please confirm that any use of statistical terms (such as trend or significant) are supported by the data, and if not please remove them. The term trend should be used only when the test for trend has been conducted.

Response: We reviewed all uses of “trend” and “significant” and ensured they are only used when supported by statistical tests. We also replaced “trend(s)” to with neutral terms such as “pattern(s)” or “variation(s)” throughout the text to avoid any potential confusion.

7. Please provide titles and legends for all figures and tables (including those in Supporting Information files).

Response: We have provided titles and legends for all figures and tables.

8. Please define all acronyms used in each figure or table in the corresponding legend.

Response: We reviewed all figures and tables to ensure that acronyms are defined within each corresponding legend. The only exception is “SPC” (subsequent primary cancer) in the y‑axis titles of Figures 2 and 3, which is defined in the figure title.

9. Please confirm that you used patient-centered language. Please note that patient-centered language is constructed with the use of post-modified nouns putting the person first in the sentence structure.

Response: We reviewed the manuscript for the use of person‑first and patient‑centered language. We retained the term “cancer survivors,” which is widely accepted in oncology and survivorship research and is often viewed as empowering. To enhance person‑first phrasing elsewhere, we replaced “younger/older survivors” with “survivors diagnosed at a younger/older age.” We believe these revisions improve clarity and consistency while maintaining alignment with field‑specific conventions.

10. When reporting age, please include ‘years’ as unit.

Response: We added “years” to all references to age.

11. Data Availability Statement: Please note that the data sharing statement following the main text and the details in online submission from do not match. Please revise. Could provide more details on the exact datasets you used in your study from SEER research data?

Response: We revised the Data Availability Statement to ensure consistency with the submission form. We also added the SEER data version in the Statement. Information about the specific SEER Research Data files used is also included in the Methods section: “… using data from the SEER (Research Resource Identifier [RRID]: SCR_006902) 8 registries (2024 submission), …”.

12. Please explain why the Supplemental incidence tables are for review purposes only? If these tables provide the number behind the graphs, we require you to make these available as part of the publication.

Response: The supplemental incidence tables were designated “for review only” in the previous submission because a reviewer had specifically requested clarification on the structure of the input data used for the age-period-cohort analyses. In this revision, we now provide all incidence data tables used as model inputs, as well as all age-period-cohort model estimates (i.e., the numerical values underlying the figures), as part of the Supporting Information.

ABSTRACT
13. Please confirm that your abstract complies with our requirements, including providing all the information relevant to this study type <https://journals.plos.org/plosmedicine/s/submission-guidelines#loc-abstract> 

Response: We reviewed the abstract against the PLOS Medicine requirements and revised it to ensure full compliance. Specifically, we added estimates and it’s 95% confidence interval in the results and a brief statement of the main study limitations.

14. Please ensure to quantify the main results (with 95% CIs and p values).

Response: We added 95% CI and p‑values to main results.

15. Please confirm that all numbers presented in the abstract are present and identical to numbers presented in the main manuscript text.

Response: We verified that all numerical values reported in the Abstract are present in the main manuscript and match exactly. To ensure full consistency, we added the sentence “SPC incidence rose from 915 per 100,000 person‑years at ages 35–39 years to 1,980 per 100,000 at ages 75–79 years among females, and from 1,228 per 100,000 to 2,945 per 100,000 across the same age groups among males” to the Results in the main text. All numbers in the Abstract now correspond directly to values reported in the main text.

16. In the abstract, please confirm you included the important dependent variables that are adjusted for in the analyses.

Response: We reviewed the abstract to ensure it includes the key dependent variables relevant to our analyses. The primary dependent variable is subsequent primary cancer (SPC) incidence, which is fully described in the abstract along with the age‑, period‑, and cohort‑specific comparisons derived from the age-period-cohort models. We stratified by sex and index cancer site. No other variables were adjusted for in the age-period-cohort models. The abstract reflects all variables central to the analysis and adheres to the journal’s requirements.

17. In the last sentence of the Abstract Methods and Findings section, please describe the main limitation(s) of the study's methodology.

Response: We added the main limitation to the end of the Methods and Findings section.

METHODS AND RESULTS
18. Please remove the numbering from the headings.

Response: We have removed all numbering from the section headings. Subheadings are now formatted in italics for better distinction.

19. Following the comment above, please revise the completed STROBE checklist and use section and paragraph numbers, e.g. “Methods, Paragraph 2”.

Response: We have updated the STROBE checklist accordingly.

20. “Input and output files are available upon request.” – please note that PLOS Medicine requires that the de-identified data underlying the specific results in a published article be made available, without restrictions on access, in a public repository or as Supporting Information at the time of article publication, provided it is legal and ethical to do so.
a) If the data are owned by a third party but freely available upon request, please note this and state the owner of the data set and contact information for data requests (web or email address). Note that a study author cannot be the contact person for the data.
b) If the data are not freely available, please describe briefly the ethical, legal, or contractual restriction that prevents you from sharing it. Please also include an appropriate contact (web or email address) for inquiries (again, this cannot be a study author).

Response: We have included the data used in this study in the Supporting Information with the revised manuscript. We have also revised the corresponding sentence in the Methods section to: Incidence data and age-period-cohort model estimates are provided in Supporting Information.,' to comply fully with PLOS Medicine policy.

21. Figure 1 requires revision:
a) We suggest specifying the x-axis label, e.g. ‘Age at index cancer (years)’.
b) We suggest specifying the x-axis label to ‘Cohort (Birth year)’ (if correct).
c) Please specify how ‘Period’ is defined.
d) Please add a unit where missing.

Response: We revised Figure 1 to incorporate all requested changes.

22. Please revise the figures according to the above comments and ensure to provide specific labels and descriptions.

Response: We revised all figures for clarity and consistency.

23. “Observed age‑specific incidence and fitted longitudinal curves were shown in Supplemental Figures” – please provide a specific reference, e.g. Figures S1 and S2.

Response: We added specific references to Figures S1 and S2.

24. Figure 1B: It looks like for women the decline plateaued between 1980 and 1985 before continuing to decline.

Response: We have revised the text to note this plateau: Among men, the decline after the peak slowed down in most recent cohorts starting in the 1970 cohort; among women, the decline continued with a plateau in the 1985 cohort (Fig 1B).

25. “Across sites of the index cancer, uterine corpus cancer survivors had the lowest SPC incidence among women, and prostate cancer survivors had the lowest incidence among men.” – At all ages? The statement doesn’t seem to be 100% accurate.

Response: We revised the statement to specify the age ranges for which it applies: “Across sites of the index cancer, uterine corpus cancer survivors had the lowest SPC incidence among women diagnosed with the index cancer between the age of 40 and 80 years, and prostate cancer survivors had the lowest incidence among men across all ages.”

26. Please confirm that where relevant figures include 95% CIs.

Response: We confirmed that all relevant figures include 95% CIs.

27. Please confirm that you specified the variables controlled for in all relevant Tables.

Response: We confirm that beyond the temporal variables inherent to the age-period-cohort analysis (age at index cancer diagnosis, calendar period, and birth cohort), no additional covariates were included in the models.

28. We still feel that the visualization of the curves could be improved, as overlapping curves are sometimes difficult to distinguish. Also, please ensure that the values behind the graphs are available in the supplementary tables.

Response: We improved curve visualization by adopting a more distinctive color palette, adding markers to point estimates, and applying lighter (30% opacity) colors to the 95% CIs to reduce overlap. All underlying values are now provided in the Supporting Information. If the editorial team prefers an alternative presentation (e.g., separate panels by cancer site), we are happy to revise accordingly.

DISCUSSION
29. Please remove the 'conclusions' subheading from the discussion. Please also remove any other subheadings from the discussion.

Response: We removed all subheadings from the Discussion.

30. “Cohort-specific SPC risks followed a bell-shaped trajectory” – Please refer to the previous comment from Reviewer #1. In your response, you indicated that you revised any such mentions.

Response: We thank the editor for pointing out the instance we missed in the previous revision. We have removed this phrasing and revised the description in the current revision (Discussion, paragraph 5).

31. “We also strengthened the framing of our contribution: our analyses provide population-level surveillance evidence that identifies survivor groups with rising or declining SPC risks, thereby informing hypotheses for future analytic studies and guiding resource allocation in survivorship care (Discussion section).” – iIt seems that you have not framed your work as inspiring hypotheses, as the reviewer suggested. Please comment.

Response: Thank you for highlighting the need to more explicitly frame our work as hypothesis‑generating. In response to the earlier reviewer comment, we revised the Discussion to remove causal language and to clarify that the age-period-cohort analyses are descriptive in nature. We also expanded our discussion of potential drivers and mechanisms underlying the observed temporal patterns, positioning these as hypotheses to be evaluated in future analytic studies.

In this revision, we further strengthened this framing throughout the Discussion to ensure that our contribution is clearly presented as population‑level surveillance evidence that identifies survivor groups with rising or declining SPC risks. We also added to the Abstract: “Main limitations include the descriptive nature of age‑period‑cohort analyses and the absence of treatment, genetic, and lifestyle data in SEER.” We hope these revisions make clear that the study is intended to motivate and inform future hypothesis‑driven research rather than to draw causal conclusions.
------------------------------------------------------------
General Journal Requests

32. We ask every co-author listed on the manuscript to fill in a contributing author statement. If any of the co-authors have not filled in the statement, we will remind them to do so when the paper is revised. If all statements are not completed in a timely fashion this could hold up the re-review process. Should there be a problem getting one of your co-authors to fill in a statement we will be in contact. YOU MUST NOT ADD OR REMOVE AUTHORS UNLESS YOU HAVE ALERTED THE EDITOR HANDLING THE MANUSCRIPT TO THE CHANGE AND THEY SPECIFICALLY HAVE AGREED TO IT.

Response: We acknowledge the requirement for all co-authors to complete the contributing author statement. We also confirm that no changes have been made to the author list.

33. Please ensure that the paper adheres to the PLOS Data Availability Policy (see <http://journals.plos.org/plosmedicine/s/data-availability>), which requires that all data underlying the study's findings be provided in a repository or as Supporting Information. For data residing with a third party, authors are required to provide instructions with contact information for obtaining the data. PLOS journals do not allow statements supported by "data not shown" or "unpublished results." For such statements, authors must provide supporting data or cite public sources that include it.

Response: We ensured full compliance with the Data Availability Policy. We have included the data used in this study in the Supporting Information with the revised manuscript.

34. Please review your reference list to ensure that it is complete and correct. If you have cited papers that have been retracted, please include the rationale for doing so in the manuscript text, or remove these references and replace them with relevant current references. Any changes to the reference list should be mentioned in the rebuttal letter that accompanies your revised manuscript.

Response: We reviewed the reference list and confirmed that no retracted papers are cited.
