# Supplementary material for: Subsequent primary cancer incidence among cancer survivors in the United States, 1975–2019: An age–period–cohort analysis
Source: PLoS Med. 2026 Apr 28;23(4):e1005034. doi: 10.1371/journal.pmed.1005034 (PMC13123941; doi:10.1371/journal.pmed.1005034)
Supplement: Attachment — Submitted filename: Response to Reviewer Comments.docx [file pmed.1005034.s007.docx]

**Response to Reviewer Comments** Manuscript “Trends in Subsequent Primary Cancer Incidence Among United States Cancer Survivors, 1975-2019: An Age-Period-Cohort Analysis" (PMEDICINE-D-25-03472R1)

We thank the editor and reviewers for their thoughtful and constructive feedback. Below, we provide detailed responses to each comment and outline the corresponding revisions made to the manuscript.

**Editor:**

1. As you will see, reviewers 2, 3 and 4 find the study of interest and find the report would be strengthened by additional information on the models and how the analyses were performed, discussion of study limitations and clarification of the advance provided by this manuscript. We further ask that you provide references to support speculation of mechanisms underlying changes in cancer recurrence over time.

Response: We have added details about the models and the analyses, discussion of limitations and implication of the study, as well as references of potential mechanisms. Please see our point-by-point responses below for details.

**Reviewer #1:**

1. This study used a large sample size of more than 3 million cancer cases to study age, period and cohort effects on second primary cancer incidence by sex. Many of the analyses were conducted combining all initial sites and then repeated stratified on the initial site of the tumor. The stated goal is to improve quality of life and reduce cancer burden among cancer survivors, however, the link between the analyses and this stated goal is ambiguous.

Response: We appreciate the reviewer’s observation regarding the need to more clearly articulate how our analyses support the stated goal. Our intention was not to claim that the APC analyses themselves directly improve quality of life, but rather that they provide essential epidemiologic evidence to inform survivorship strategies, including surveillance and intervention of known risk factors based on the literature. We have revised the manuscript to strengthen this link.

Specifically, we now emphasize that population‑level surveillance of SPC incidence is a prerequisite for designing effective survivorship care. By identifying age, period, and cohort patterns, our analyses highlight which survivor groups (e.g., older adults, recent cohorts of female lung cancer survivors, male bladder cancer survivors) are experiencing rising SPC risks. These findings can guide risk‑stratified surveillance and prevention efforts, such as tailoring screening guidelines, prioritizing smoking cessation or UV protection interventions, and allocating survivorship resources to groups with increasing burden. Understanding temporal dynamics of SPC risk also informs projections of future cancer burden, which is critical for health system planning and policy development aimed at reducing morbidity and improving survivor quality of life.

We have revised the Discussion (section 4.4) to explicitly state how the descriptive APC results translate into actionable insights for survivorship care and cancer control. We also added references to survivorship guidelines and prevention strategies that can be informed by these epidemiologic trends.

2. Another reasonable possible approach would be to identify patterns in second primary cancers by site of second cancer, particularly if one was interested in identifying risk-based screening opportunities. The interpretation of the site specific second primary data does not clearly lead to public health opportunities.

Response: We thank the reviewer for this insightful suggestion. We agree that identifying patterns by site of the second primary cancer could provide additional opportunities for risk‑based screening and prevention. Our current analysis characterizes SPC incidence by index cancer site, which provides a foundational overview of survivor groups and has implications for surveillance and survivorship care. For example, rising SPC incidence among female lung cancer survivors underscores the importance of surveillance and screening for subsequent cancers and smoking cessation in this population. Similarly, the increasing risks among skin melanoma survivors highlight the need for dermatologic surveillance and UV protection strategies. Our findings provide a population‑level foundation for such targeted investigations. We have revised the Discussion (section 4.2 and 4.4) to clarify these implications and to note that future studies can further inform targeted screening strategies by examining SPCs by site of the subsequent cancer. We also added examples of how our findings already suggest public health opportunities, such as intensified surveillance for female lung cancer survivors and dermatologic follow‑up for melanoma survivors.

3. Overall, some linguistic issues diminish the impact of this work. In addition, the author claims they have 'disentangled' age, period and cohort effects and this is unclear.

Response: We thank the reviewer for noting the need to improve clarity in language. We have revised the manuscript to improve phrasing and readability throughout, and we have clarified our description of the age‑period‑cohort (APC) modeling approach. Specifically, we replaced the term “disentangled” with more precise language, stating that the APC model decomposes observed incidence trends into age, period, and cohort components under the Rosenberg method.

4. The statement in results (paragraph 1) that 'they represent 64% and 63% of all cancer survivors among women and men in the study period is not clear. Given that approximately 500K SPCs were detected among 3.7M survivors, these numbers seem incorrect, which suggests some specificity is lacking in this sentence. Similar ambiguity of phrasing occurs in referencing Figure 2 for the first time (Results paragraph 2), where authors state that age-related increases were observed among survivors of 'the most index cancer sites'.

Response: We thank the reviewer for pointing out the ambiguity in our phrasing regarding the proportion of survivors and the description of Figure 2. We have revised the statement to “Collectively, the five most common index cancer sites accounted for 64% of female survivors and 63% of male survivors in the sample.”

In referencing Figure 2, we have revised the description to indicate specific cancer sites. Now it reads “When stratified by the site of the index cancer, the general increasing pattern with age was observed among survivors of four of the five most common index cancer sites among women (lung/bronchus, colon/rectum, uterine corpus, melanoma) and all five most common index cancer sites among men (prostate, lung/bronchus, colon/rectum, bladder, melanoma) (Fig 2). An exception was among female breast cancer survivors, where SPC incidence showed much slighter variations with a nadir of 1,328 per 100,000 person-years when diagnosed at ages 40–45.” This ensures readers understand exactly which sites are being discussed.

5. In describing the temporal trends, the authors describe 'a bell curve' which is a distribution within a population and not over time. This descriptor of the morphology of the curve is confusing. Similarly the term, n-shaped patterns was used, without definition.

Response: We thank the reviewer for noting that our use of terms such as “bell curve” and “n‑shaped” may be confusing to readers. We have revised the Results section to replace these descriptors with more precise language. Specifically, we now describe the cohort‑specific SPC risk as “When all sites are considered, the risks of SPC for cohorts born between 1885 to 1995 peaked at the 1925 and 1930 cohorts among men and the 1935 cohort among women and declined in later cohorts. Among men, the decline after the peak slowed down in most recent cohorts starting in the 1970 cohort; among women, the decline continued (Fig 1B).” We now describe “n‑shaped” patterns as “trajectories that rose in earlier cohorts, peaked among cohorts born between 1935 and 1945, and then declined in more recent cohorts.” (Section 3.2).

6. The observation of lung, bladder and skin having patterns with age is attributed to tobacco smoke and UV exposure without justification (discussion).
   Response: We thank the reviewer for highlighting the need to avoid over‑interpretation of descriptive patterns. We have revised the Discussion to clarify that the observed age‑related SPC patterns in lung, bladder, and skin cancer survivors is consistent with known carcinogenic exposures (tobacco, UV radiation) and patterns of SPC frequencies among survivors of these index cancers, but we do not claim direct attribution. We added references to prior studies documenting these associations as follows (Section 4.3):

“Prior studies indicate that subsequent primaries in lung and bladder cancer survivors often include smoking‑related cancers [13,48], while subsequent primaries in melanoma survivors are dominated by melanomas, consistent with cumulative ultraviolet radiation exposure [49,50]. Although our descriptive analyses cannot establish causality, these patterns align with known carcinogenic exposures and highlight the importance of tailoring surveillance and prevention strategies to the index cancer. For example, smoking cessation and lung/bladder cancer follow‑up, UV protection and dermatologic surveillance for melanoma survivors may represent opportunities to mitigate SPC burden.”

7. Patterns of colorectal cancer and breast cancer in those diagnosed before 40 was cited as evidence of the need for early-life survivorship interventions (which seems unwarranted given the likely contribution of germline genetics to early onset breast and colorectal cancers, and possibly second primaries).

Response: We agree that germline genetics (e.g., BRCA mutations, Lynch syndrome) likely play a major role in early‑onset breast cancer and colorectal cancer and subsequent primaries. We have revised the text to emphasize that these findings underscore the need for genetic counseling and tailored surveillance in young survivors, rather than suggesting early‑life survivorship interventions more broadly. Nonetheless, research has pointed to the role of lifestyle factors, such as obesity and sedentary lifestyle, in the rise of breast cancer and colorectal cancer among younger individuals. We have revised the Discussion as follows:

“A noteworthy observation is that breast cancer (in women) and colorectal cancer (in men) diagnosed before age 40 had the highest SPC risk among the most common index cancer sites studied. In addition to the well documented role of germline predisposition in early-onset breast and colorectal cancers [32, 36, 37], recent studies suggest that obesity and sedentary lifestyle may contribute to rising incidence in younger populations [16, 38]. Our finding highlights the importance of integrating genetic counseling with lifestyle interventions into risk-stratified surveillance, particularly given that these cancers are among the fastest-growing malignancies in young Americans [23, 34].”

8. Declines over calendar period were attributed to detection, diagnosis, treatment, and reduced exposure and enhanced survivorship care. This speculative interpretation of the data does not substantially improve our appreciation for the dynamics of cancer prevention and control. In limitations, the authors acknowledge that the work inspires hypotheses (rather than clearly providing explanations), but the overall impact of the analyses remains unclear.

Response: We thank the reviewer for highlighting the need to avoid speculative attribution of calendar‑period declines. We have revised the Discussion to clarify that our APC analyses are descriptive and cannot establish causality. We now state that the observed declines are consistent with improvements in detection, treatment, reduced exposures, and enhanced survivorship care, but future research is needed to clarify their roles. We also strengthened the framing of our contribution: our analyses provide population‑level surveillance evidence that identifies survivor groups with rising or declining SPC risks, thereby informing hypotheses for future analytic studies and guiding resource allocation in survivorship care (Discussion section).

9. Visualization of the curves are small and not well-delineated with color and the site specific analyses are not interpreted in detail with respect to treatment-related exposures or other co-exposures. These gaps limit the ability of the paper to advance SPC prevention strategies.

Response: We thank the reviewer for pointing out the need for clearer visualization and more detailed interpretation of site‑specific analyses. We have enlarged the figures and revised the colors to improve readability.

In the Discussion, we now provide more detailed interpretation of site‑specific SPC patterns in relation to treatment exposures and co‑exposures. For example, elevated SPC risks among bladder and lung cancer survivors may reflect the combined impact of smoking and treatment‑related carcinogenicity, while melanoma survivors face ongoing risk of subsequent melanomas due to cumulative UV exposure. We also note that early‑onset breast and colorectal cancer survivors often have germline predispositions, underscoring the importance of genetic counseling. While our registry‑based analyses cannot directly measure treatment exposures, these contextual interpretations help situate our findings within the broader landscape of SPC prevention and survivorship care.

**Reviewer #2:**

OVERVIEW

This is an interesting and novel application of age-period-cohort analysis to characterize incidence rates of second primary cancers (SPCs) in an open cohort of cancer survivors. The cohort under observation is persons living in the SEER-8 catchment areas between 1975 - 2019, a population that includes roughly 10% of the total US population.
SEER-8 is the longest running nationally authoritative cancer registry in the US with almost 50 years of follow up. For this reason, it is optimal for obtaining a historical perspective on long-term cancer trends in the US, especially among non-Hispanic white men and women.
This historical perspective covers six social generations in the US. For adult cancers, from the Lost Generation through Generation X, and for childhood cancers, the Millennials.
The authors arrived at several useful take-home messages. Notably, they report that SPC risks increased in more recent cohorts of female survivors of lung and bronchus cancer and male survivors of urinary bladder cancer (Figure 3).
This report also highlights that much progress has been made, broadly speaking, since the peak of SPC susceptibility in persons born circa 1935 (Figure 1B). Having said that, it is also sobering to see evidence that the secular declines appear to have slowed since circa 2000 (Figure 1C).

Response: We thank the reviewer for their thoughtful summary of our manuscript and for recognizing the novelty of applying age‑period‑cohort analysis to SPC incidence using SEER‑8 data. We appreciate the emphasis on the historical perspective and generational framing, which we have highlighted more clearly in the revised Discussion and Conclusion.

MAIN COMMENTS

Basically, two suggestions.

1. The overall analysis approach is sound and demonstrates a nice application of what might be called  'descriptive' APC analysis. The authors' account of what they did is correct, but I would like to see more details about how the appropriate 'Lexis diagrams' were constructed using the SEER program data. (First suggestion).
This reviewer is not 'hands-on' when it comes to using SEER*Stat! Can the authors' simply select second primary cancers only, and then conduct a rates session to obtain the appropriate tables of person-years at risk and corresponding numbers of events? Or, do you need to conduct a case listing session of cancer survivors so that these individuals can be censored at death? If the explanation is non-trivial, perhaps this material could be included in an online supplement. This might benefit other investigators.

Response: We thank the reviewer for recognizing the soundness of our APC approach and for suggesting greater detail on how Lexis diagrams were constructed using SEER data. Specifically, we obtained the number of subsequent primary cancer events and person‑years at risk via MP‑SIR (Multiple Primary–Standardized Incidence Ratio) sessions in SEER*Stat, stratified by 5‑year age groups and 5‑year calendar periods of diagnosis. The MP‑SIR session is designed specifically for analysis of multiple primary cancers. Person‑years at risk were censored at death or end of follow‑up. We then used this information as input for the APC web tool. We have added these details to the Methods section (Section 2.2), and we believe this additional transparency will benefit other investigators interested in replicating descriptive APC analyses of SPC incidence.

2. Considering the longitudinal age incidence curves shown in Figure 1A, 2A, and 2B: These are very high rates. For example, in Figure 1A, above 2% per year among men over 60 and 1.5% per year among women over 60. To me this begs the question, how do the longitudinal age curves in Figure 1 compare with the corresponding longitudinal age curves for first malignancies obtained from the same SEER-8 population? (Second suggestions). Are we talking about a 50% increase, or more?
One reason for adding this information is it provides a nice quality-control check for the data assembly stage (first suggestion).
FWIW, such a comparative analysis technically describes the relative experience of the reference 1915 cohort, but in light of Figure 1B, these absolute curves are equally descriptive for the 1940 male birth cohort and the 1955 female cohorts.
This reviewer recognizes that extending such an analysis to every curve in Figure 2 goes beyond the scope of the present work, but perhaps merits mention as a possible avenue for future studies.

Response: We thank the reviewer for highlighting the value of comparing longitudinal age‑specific SPC incidence curves with those for first malignancies in the same SEER‑8 population. We agree that such comparisons provide important context for interpreting the magnitude of SPC risk and serve as a useful quality‑control check. We have included age‑specific SPC incidence rates and expected incidence rates (i.e., the incidence in the general population) with this resubmission. In SEER*Stat, expected event counts were derived by applying general population incidence rates to the accumulated person‑years of follow‑up. The tables also present the number of SPC events and person-years of follow-up for transparency (see the supplemental file for reviewer only). These rates are comparable to published population‑based estimates, confirming the accuracy of our data assembly. For example, the incidence of first malignancies in 2010 was approximately 900 per 100,000 among 50–64‑year‑old males and 2,700 per 100,000 (i.e., 2.7%) among males aged 65+, and approximately 800 per 100,000 among 50–64‑year‑old females and 1,750 per 100,000 among females aged 65+ (Siegel et al., Cancer Statistics, 2025; <https://acsjournals.onlinelibrary.wiley.com/doi/10.3322/caac.21871>). Incidence was ~1700/100,000 among 70-84-year-old females and ~2400/100,000 among 70-84-year-old males in 2015 (Kehm et al., <https://pmc.ncbi.nlm.nih.gov/articles/PMC6686848/>). Moreover, prior studies have shown that cancer survivors experience a 20–30% higher incidence of subsequent primary cancers compared to the general population. We have also added text in the Discussion noting that systematic comparisons across cancer sites represent an important avenue for future research.

Minor Comment:

3. Reference cohort in Fig 1 is also 1915, not 1885 as stated in the Figure 1 legend.

Response: We thank the reviewer for catching this error. The reference cohort in Figure 1 is indeed 1915, not 1885 as originally stated in the legend. We have corrected the figure legend accordingly in the revised manuscript.
**Reviewer #3**: Statistical review
This paper reports an analysis of prevalence of cancer recurrence and how this has changed over time, between the sexes and in different cancer types. I had some comments on the statistical methods and reporting, which are below.
1.      Abstract: I am sympathetic about summarising a large number of results in an abstract. However, having some quantitative results for some of the statements would be useful, e.g. 'increased with age..', 'steeper rises among men'.

Response: We thank the reviewer for this helpful suggestion. We have revised the abstract to include representative quantitative results to anchor key statements. The sentence now reads “… SPC incidence increased with age at index cancer diagnosis, rising among females from 915 per 100,000 person‑years at ages 35-39 to 1,980 per 100,000 at ages 75-79, and among males from 1,228 per 100,000 to 2,945 per 100,000 across the same age groups, demonstrating steeper rises in men.”

2.      "Cohort-specific SPC risk peaked in the 1935-1945 birth cohorts and declined in later cohorts, except among female survivors of lung cancer and male survivors of bladder cancer, where risks continued to rise." - when I initially read this, it sounded like selected results from a large number of potential combinations. I don't think it's as bad as I initially thought due to there only being five cancer sites considered (although the number of combinations with age, sex etc is higher). Perhaps as a discussion point, were there any multiplicity concerns here?

**Response:** We appreciate the reviewer’s thoughtful comment regarding multiplicity. Each site‑specific analysis was conducted within a distinct survivor population, so these results do not represent multiple tests of the same cohort. Our APC analyses are descriptive in nature, intended to highlight broad population‑level patterns rather than to conduct formal hypothesis testing. Accordingly, we did not apply multiplicity adjustments. Nonetheless, we agree that examining multiple strata across site, sex, age, and cohort increases the potential for over‑interpretation. We have added the following text in the Limitation (Section 4.5) to acknowledge this limitation and to emphasize that our findings should be interpreted cautiously.

“Because our APC analyses are descriptive and each site‑specific analysis involves a distinct survivor population, we did not apply formal multiplicity adjustments. Nonetheless, over‑interpretation is possible given the large number of age-, period-, and cohort-strata.”

3.      Statistical analysis: it would be useful to mention that the modelling technique allows quantification of uncertainty (e.g. via confidence intervals).

Response: We thank the reviewer for this helpful suggestion. Our APC modeling framework does provide quantification of uncertainty via confidence intervals. We have clarified this in the Methods of the revised manuscript: “All APC model estimates were accompanied by 95% confidence intervals to quantify statistical uncertainty.”

4.      Statistical analysis: my understanding of the models used is not good. Is this correct: a person's follow-up would start at diagnosis of the index cancer and then the number of SPCs following is modelled as a Poisson outcome. Is the follow-up length included via an offset like would happen in a Poisson regression? If someone died of the index cancer, would the follow-up be truncated at death? Does this potentially ignore the potential for someone to have a SPC prior to death that is undiagnosed? I think including more intuition on the approach might be useful, together with any assumptions (and drawbacks in the discussion).

Response: We thank the reviewer for raising these questions. Follow‑up begins at the time of index cancer diagnosis and is truncated at death or end of follow‑up, consistent with SEER ascertainment. Subsequent primary cancers are modeled as Poisson outcomes, with person‑time included via a log offset to account for varying follow‑up times. Parameters are estimated using weighted least squares, allowing for extra‑Poisson variation to account for overdispersion in the data. This modeling approach reflects the fact that real‑world incidence data often exhibit greater variability than expected under a strict Poisson process. We have added this information in the revised manuscript (Section 2.2)

We acknowledge that SPCs occurring but not diagnosed prior to death are not captured, which may lead to under‑ascertainment in older or more aggressive cancers. However, SEER data undergo thorough review by trained registrars and a rigorous validation process. Moreover, SEER is one of the largest and longest‑running population‑based cancer registries in the US, which makes this type of analysis possible. We have added this clarifying text in the Limitations: “It is also possible that SPCs that occur but remain undiagnosed prior to death are not captured in SEER, which may lead to under‑estimation of SPC incidence in certain populations.”

5.      Statistical analysis: for the five different cancer sites, was this a stratified analysis using only data from people with that cancer site, or was a model with parameters for the cancer site used?

Response: We thank the reviewer for raising this important clarification. The analyses for the five cancer sites were conducted as stratified analyses, using only data from individuals diagnosed with the specific index cancer. In other words, each site was analyzed separately within its own survivor population, rather than fitting a single pooled model with site parameters. We have clarified this in the Methods section to ensure transparency.

6.      Statistical analysis: Because age, period, and cohort effects are linearly dependent (cohort = period − age), it might be helpful if the authors briefly note how the Rosenberg method addresses the identifiability issue and what constraints are imposed. This would reassure readers that the results reflect interpretable effects.

Response: We thank the reviewer for raising this important point. As the reviewer notes, age, period, and cohort effects are linearly dependent. The Rosenberg method addresses this identifiability issue by estimating uniquely defined functions of the parameters, such as net drift, longitudinal age trends, and period/cohort deviations, rather than attempting to estimate all three effects simultaneously. We have added the following text in the Methods to clarify how the Rosenberg method resolves the identifiability problem and what constraints are imposed:

“In this study, age-period-cohort analyses were conducted using the Rosenberg method [23]. The Rosenberg approach resolves the identifiability problem (i.e., age, period, and cohort effects are linearly dependent) by estimating uniquely defined functions of the parameters. These include net drift (overall annual percentage change), longitudinal age trends, and period and cohort deviations. Linear trends are absorbed into the net drift, while deviations are constrained to sum to zero and decomposed into orthogonal quadratic components, ensuring interpretability. Cohort deviations are weighted to account for the variable number of periods each cohort is observed. Parameters were estimated using weighted least squares under the assumption that SPC counts follow a Poisson distribution. Person‑years at risk were included via a log offset to account for varying follow‑up times, and extra‑Poisson variation was incorporated to account for overdispersion in the data [23].”

7.      Results: Some indication of model fit (e.g. comparison of observed vs. fitted incidence rates, or summary of overdispersion parameters) would strengthen confidence in the results and clarify whether the APC model adequately captured the main features of the data.

Response: We appreciate this suggestion. The Rosenberg method is designed to bypass the identifiability problem by estimating functions rather than parameters. Because of this, conventional goodness‑of‑fit statistics tied to full parameter estimation are not available. Instead, the emphasis is on interpretability and reproducibility of fitted functions. To help assess the fit, we have added overlays of observed and fitted age‑specific incidence rates for the earliest, middle, and latest periods in the supplemental material (Figures S1 and S2). Observed incidence (dots) closely paralleled fitted longitudinal curves (dashed lines), indicating that the APC model captured the main features of the data. We have added the following text in the Results: “Observed age‑specific incidence and fitted longitudinal curves were shown in Supplemental Figures. The parallel shapes suggest that the APC model adequately captured the main features of the data.” (Section 3.1)

**Reviewer #4**

This is a very well written paper describing the risks of subsequent primary cancers (SPCs) among survivors of first primary cancers using data from 8 SEER registries. Strengths of the paper include the very large number of primary cancers (3.36 million) and >500,000 SPCs. This has allowed for robust analyses to investigate age, period and cohort effects in the 5 most common cancers in women and men. The results are presented clearly and are interpreted appropriately. Numbers of cancer survivors are growing around the world and warrant careful monitoring of risks and trends in SPCs. As the authors conclude, patterns of SPC risk are complex. The paper makes a valuable contribution to our understanding.

Response: We thank the reviewer for the encouraging comments and recognition of our study’s strengths. We agree that monitoring SPC risks is increasingly important as the survivor population grows worldwide, and we appreciate the acknowledgment that our findings contribute meaningfully to understanding these complex patterns.

1. The data come from only 8 SEER registries which suggests that some primary and SPCs may have been missed if diagnosed in people who moved into or out of the areas covered by these registries. This limitation and its implications should be discussed. Discussion of the quality of the data and any changes in the study period from 1975 to 2022 would also be warranted.

Response: We thank the reviewer for raising this important point. We have expanded the Discussion to acknowledge that our analyses were restricted to 8 SEER registries. Individuals who migrated out of registry catchment areas were censored at the time of migration, so although incidence estimates remain valid for the follow‑up period they contributed, SPCs diagnosed after migration would not be captured, which may reduce case counts and slightly limit precision. We now note this limitation explicitly (Section 4.5).

We also added text on data quality and changes over time. SEER registries are widely recognized for their high standards of case ascertainment and follow‑up, with consistent quality control procedures throughout the study period. Nonetheless, improvements in diagnostic practices, coding, and registry coverage since 1975 may have influenced SPC detection, particularly in the earlier years. We have added these in the Limitation section (Section 4.5).

2. As a minor point, there is variable formatting of the references in the text (with or without a superscript). There is a typographical error in the footnote to table 1 (fond).

Response: We thank the reviewer for noting these issues. We standardized reference formatting throughout the manuscript and corrected the typo in the footnote to Table 1.
